# Hemp seed significantly modulates the endocannabinoidome and produces beneficial metabolic effects with improved intestinal barrier function and decreased inflammation in mice under a high-fat, high-sucrose diet as compared with linseed

**DOI:** 10.3389/fimmu.2022.882455

**Published:** 2022-09-26

**Authors:** Rim Ben Necib, Claudia Manca, Sébastien Lacroix, Cyril Martin, Nicolas Flamand, Vincenzo Di Marzo, Cristoforo Silvestri

**Affiliations:** ^1^ Centre De Recherche De l’Institut Universitaire De Cardiologie Et De Pneumologie De Québec (IUCPQ), Quebec, QC, Canada; ^2^ Département De Médecine, Faculté de Médecine, Université Laval, Quebec, QC, Canada; ^3^ Institut Sur La Nutrition Et Les Aliments Fonctionnels (INAF), Quebec, QC, Canada; ^4^ Canada Research Excellence Chair in the Microbiome-Endocannabinoidome Axis in Metabolic Health (CERC-MEND), Quebec, QC, Canada; ^5^ École de nutrition, Faculté Des Sciences De l’Agriculture Et De l’Alimentation (FSAA), Université Laval, Quebec, QC, Canada; ^6^ Centre Nutrition, Santé et Société (NUTRISS), Université Laval, Quebec, QC, Canada

**Keywords:** endocannabinoidome, gut microbiome, hemp seeds, linseeds, omega-3, nutrition, obesity

## Abstract

Omega-3 fatty acids support cardiometabolic health and reduce chronic low-grade inflammation. These fatty acids may impart their health benefits partly by modulating the endocannabinoidome and the gut microbiome, both of which are key regulators of metabolism and the inflammatory response. Whole hemp seeds (*Cannabis sativa*) are of exceptional nutritional value, being rich in omega-3 fatty acids. We assessed the effects of dietary substitution (equivalent to about 2 tablespoons of seeds a day for humans) of whole hemp seeds in comparison with whole linseeds in a diet-induced obesity mouse model and determined their effects on obesity and the gut microbiome-endocannabinoidome axis. We show that whole hemp seed substitution did not affect weigh gain, adiposity, or food intake, whereas linseed substitution did, in association with higher fasting glucose levels, greater insulin release during an oral glucose tolerance test, and higher levels of liver triglycerides than controls. Furthermore, hemp seed substitution mitigated diet-induced obesity-associated increases in intestinal permeability and circulating PAI-1 levels, while having no effects on markers of inflammation in epididymal adipose tissue, which were, however, increased in mice fed linseeds. Both hemp seeds and linseeds were able to modify the expression of several endocannabinoidome genes and markedly increased the levels of several omega-3 fatty acid–derived endocannabinoidome bioactive lipids with previously suggested anti-inflammatory actions in a tissue specific manner, despite the relatively low level of seed substitution. While neither diet markedly modified the gut microbiome, mice on the hemp seed diet had higher abundance of *Clostridiaceae 1* and *Rikenellaceae* than mice fed linseed or control diet, respectively. Thus, hemp seed-containing foods might represent a source of healthy fats that are not likely to exacerbate the metabolic consequences of obesogenic diets while producing intestinal permeability protective effects and some anti-inflammatory actions.

## 1 Introduction

Omega-3 fatty acids (FAs) are found in seafood, leafy vegetables, as well as in nuts and seeds such as linseeds and hemp seeds, being derived from elongation and desaturation of alpha-linolenic acid (ALA) (18:3 ω-3) (1). Historically, the ratio of dietary omega-6 to omega-3 FAs was close to 1, whereas, today, it has increased to 20:1 in Western diets, making it at odds with the diet on which human beings evolved (2). Indeed, several nutritional studies show that a high omega-6/omega-3 FA ratio has a direct link to the “obesity epidemic,” (3, 4) with an increasing number of studies supporting the cardiometabolic health benefits of a diet rich in omega-3 FAs. Most studies focus on eicosapentaenoic acid (EPA) and docosahexaenoic acid (DHA), indicating that these FAs are linked to a lower risk of developing various aspects of the metabolic syndrome and cardiovascular disease (5, 6). These benefits are suggested to be linked to anti-inflammatory activity resulting from the displacement of the pro-inflammatory, omega-6 FA arachidonic acid from phospholipids membranes (7, 8) and downregulation of the inflammasome in various tissue, including adipose tissue (9). It also appears that omega-3 FAs exert part of their health benefits by modulating the endocannabinoid system (ECS) and the gut microbiome, both of which are key regulators of several aspects of cardiometabolic health and energy metabolism.

The ECS plays a pivotal role at both central and peripheral levels to generally decrease metabolism and increase energy storage in several organs (10) as well as decrease inflammation (11). Endocannabinoids (ECs; *N*-arachidonoylethanolamine [AEA] and 2-arachidonoylglycerol [2-AG]) are long-chain arachidonic acid (AA)–derived signaling lipids generally produced on demand from phospholipid precursors (12) and originally found to activate cannabinoid receptors type 1 and type 2 (CB1 and CB2) (13). The modification of the omega-6/omega-3 ratio modulates the levels of AEA and 2-AG as well as the levels of related lipid signaling mediators that make up the expanded ECS, or endocannabinoidome (eCBome), which is composed of AEA and 2-AG and their endogenous congeners (the *N*-acylethanolamines [NAEs] and 2-monoacylglycerols [2-MAGs], respectively, which can contain various omega-3, -6, and -9 unsaturated FAs as well as saturated FAs) and other analogues, their several receptors and metabolic enzymes (14). An obesogenic diet rich in omega-6 increases AEA and/or 2-AG tissue concentrations and signaling (15–18) due primarily to an increase in the levels of esterified AA (19) and changes in the expression of their anabolic and catabolic enzymes (20) as well as an increase in CB1 receptor levels (16). Conversely, an increased consumption of omega-3 FAs decreases the levels of these ECs and increases the levels of the DHA- and EPA-containing NAEs, that is, *N*-docosahexaenoylethanolamine (DHEA) and *N*-eicosapentaenoylethanolamine (EPEA), in conjunction with improved metabolic parameters in rodents and humans (19, 21–24). Thus, it is surmised that the positive health effects of long-chain omega-3 FAs derive, at least in part, from the modulation of the ECS (25, 26). Furthermore, enhancement of the tissue concentrations of omega-3-derived NAEs (and possibly 2-acylglycerols), which have decreased affinity for CB1 receptors and exert anti-inflammatory actions *via* a regulation of pro-inflammatory cytokine and nitrous oxide levels (27), possibly also by being converted into anti-inflammatory epoxides (28), has been postulated as a potential mechanism for some of the actions of dietary omega-3 FAs (7).

The microbiome is directly modulated by dietary changes, with obesogenic diets linked to dysbiosis and a decreased bacterial diversity (29). Furthermore, the study from Pu S. and colleagues, showed that serum lipopolysaccharide (LPS) levels were higher in mice fed with lard compared with those fed with fish oil, indicating that microbial factors are present in the periphery contributing to white adipose tissue inflammation (30). By modifying the diet with supplementation with omega-3 FAs from fish oil and krill oil, the gut microbiota composition can be restored (31). Fish oil consumption increases relative abundance of *Akkermansia muciniphila*, which is a mucin-degrading, gram-negative bacterium inversely correlated with overweight, obesity, and diabetes, in both murine (32) and human (33) studies, and has been suggested to exert beneficial immune-modulatory actions (34).

Recently, a bidirectional interaction between the gut microbiome and the ECS has come to light that is relevant to metabolic health (35). The eCBome changes, such as increased CB1 expression and 2-AG levels, accompanying an obesogenic diet are associated with an altered microbiome (36, 37). Furthermore, germ-free mice show many changes within the eCBome, several of which are reversed with reintroduction of the microbiome in to the gut (38), whereas desensitization of CB1 within a genetic model that harbors increased 2-AG levels prevents diet-induced obesity in conjunction with specific alterations to the microbiome (39). The CB1 receptor and eCBome bioactive lipids are involved in regulating intestinal permeability through the regulation of tight junction levels, with increased CB1 activity increasing permeability, allowing inflammatory molecules (such as LPS) to cross the epithelial cells of the intestine and reach the bloodstream (37). In the same way, the gut microbiome changes induced by an obesogenic diet increase gut barrier permeability, thereby causing increased circulating levels of LPS, which, in turn, modulate the eCBome (40).

Hemp seeds (*Cannabis sativa*) represent a potentially important food crop for individuals with low consumption rates of omega-3 FAs and fiber. Whole hemp seeds are very rich in essential fatty acids (EFAs) and other polyunsaturated fatty acids (PUFAs), including the omega-6 FA linoleic acid (LA) and the omega-3 FAs ALA and stearidonic acid (SDA), which can more easily be converted to EPA (41, 42). This latter point may explain, in part, why daily supplementation with 30 ml of hemp seed oil was found to produce positive cardiometabolic effects in humans (43).

On the basis of all the above, we set out to examine the effects of whole hemp seeds on the development of obesity, insulin resistance, and various metabolic and inflammatory parameters in a high-fat/high-carbohydrate diet-induced obesity murine model and to correlate the observed changes to concurrent modifications of the eCBome and gut microbiota. We compared the effects of whole hemp seeds with whole linseeds, which have already been investigated in this context (44).

## 2 Materials and methods

### 2.1 Animals, housing, and diets

All studies were carried out at “Institut Universitaire de Cardiologie et Pneumologie de Québec” (IUCPQ, QC, Canada). Six-week-old male C57BL/6J (48 mice) were divided randomly into four groups (**Supplementary Figure 1**) after 1 week of acclimatization to the animal facility and access to regular chow diet. All the mice were provided with water ad libitum and housed individually on a regulated daylight cycle. For the experimental protocol, the control group was fed a diet containing 10% fat, 20% protein, and 70% carbohydrates (LFLS). All other groups were fed an obesogenic high-fat/high-sucrose (HFHS) diet composed of 45% lipids, 20% protein, and 35% carbohydrates without or with the substitution of whole linseeds (Lin group) or whole hempseeds (Hemp group) (**Supplementary Figure 2**). Fifteen percent of the fat intake in the substituted diets was derived from the seeds. The quantity of seeds supplemented was determined first, based on a study by Demizieux et al., which showed that this rate is sufficient to obtain a beneficial effect for Linseeds (21), and second, on the idea that we can mimic a clinical study with 15% of fat coming from seed, because it represents 37 g of whole Finola hemp seeds and that can easily be consumed daily. The hempseeds (Finola) were provided by Nature’s Decision, and the different diets were made by Research Diet USA. The study was approved by the Institutional Review Board of the Laval University for the protection of animals (license #2018101-1).

Body weight and food intake were assessed twice a week. Body composition (lean mass, fat mass, and water content) was determined by nuclear magnetic resonance (NMR) using a ^C^Bruker’s Minispec Analyzer at weeks 0 and 8. The liver weights of animals were determined after sacrifice.

### 2.2 Oral glucose tolerance test and intestinal permeability assay

After 8 weeks on the diet, mice were fasted 6 h prior to an oral glucose-tolerance test (OGTT). A total of 2 µl/g of 50% dextrose solution was administrated by gavage, and blood glucose was measured from the tail at 0, 15, 30, 60, 90, and 120 min. Blood samples were collected during all-time points for insulin determination; measured with the ultrasensitive mouse ELISA kit (ALPCO). The HOMA-IR was determined using the following formula: area under the curve of fasting insulinaemia (ng/ml) x area under the curve of fasting glycemia (mg/dl).

During the OGTT, we evaluated the intestinal permeability by the measurement of sulfonic acid fluorescence. Sulfonic acid solution (150 µl) (mix 1.5-mg sulfonic acid with 150 µl of 0.5% carboxymethylcellulose sodium salt [CMC] for each mouse) were administered with the dextrose solution. Plasma (5 µl) was pipetted, each well of a black 96-well plate with optical bottom placed on ice. Before reading the plate, 150 µl of 0.5% CMC was added to each well and the content was mixed by pipetting. The plate was read on a plate reader with excitation/emission 485/528 nm.

### 2.3 Tissue collection and measurement of circulating factors

Mice were sacrificed 1 week after the OGTT to eliminate possible effects of stress on the eCBome analysis. After 12 h of fasting, intracardiac blood samples were taken in tubes, which contain EDTA (K3) from animals during deep isoflurane anesthesia followed by cervical dislocation. Then, mice were dissected to collect tissues, including liver, muscle, and adipose tissue. Plasma levels of cholesterol, triglycerides, and HDL cholesterol were measured by the biochemical analysis platform of the Quebec Heart and Lung Institute by colorimetry (Siemens Dimension Vista1500). Ghrelin, GIP, GLP-1, insulin, glucagon, resistin, IL-6, TNF-α, adiponectin, leptin, IFN-γ, IL-17, IL-10, IL-1β, and PAI-1 were assessed *via* Bio-Rad bioplex assays (Bio-Plex Pro™ Mouse Diabetes Standard 8-Plex+Adiponectin, and Bio-Plex Pro™ Mouse Cytokine Standard 23-Plex, Group I).

### 2.4 Liver triglyceride measurement

To measure the hepatic triglyceride levels, 50 mg of liver were used for a standard chloroform-methanol Folch lipid extraction as previously described (45), and triglycerides were measured by commercial kit (Randox Laboratories, Crumlin, UK).

### 2.5 Analysis of eCBome mediators by HPLC-MS/MS

Lipids were extracted from tissue samples according to the Bligh and Dyer method (46) with slight modifications. Tissues were processed and analyzed randomly and blindly. Briefly, the samples of each mouse were powdered in liquid nitrogen, and about 10 mg was homogenized in 1 ml of a 1:1 Tris-HCl 50 mM (pH 7): methanol solution containing 0.1M acetic acid and 5 ng of deuterated standards. The hypothalamus followed the same steps but were directly homogenized in the 1:1 Tris-HCl solution with a tissue grinder and not powered in liquid nitrogen. Chloroform (1 ml) was then added to each sample, which was then vortexed for 30 s and centrifuged at 3,000*g* for 5 min. The organic phase was collected, and another 1 ml of chloroform was added to the inorganic one. This was repeated twice to ensure the maximum collection of the organic phase. The organic phases were pooled and evaporated under a stream of nitrogen and then suspended in 50 µl of mobile phase containing 50% of solvent A (water + 1mM ammonium acetate + 0.05% acetic acid) and 50% of solvent B (acetonitrile/water95/5 + 1 mM ammonium acetate + 0.05% acetic acid). Each sample (40 μl) was finally injected onto an HPLC column (Kinetex C8, 150 × 2.1 mm, 2.6 μm, Phenomenex) and eluted at a flow rate of 400 μl/min using a discontinuous gradient of solvents A and B (47). The quantification of eCBome-related mediators (**Supplementary Table 1**) was carried out by HPLC system interfaced with the electrospray source of a Shimadzu 8050 triple quadrupole mass spectrometer and using multiple reactions monitoring in positive ion mode for the compounds and their deuterated homologs.

In the case of unsaturated monoacyl-glycerols, the data are presented as 2-monoacylglycerols (2-MAGs) but represent the combined signals from the 2- and 1 (3)-isomers, because the latter are most likely generated from the former *via* acyl migration from the sn-2 to the sn-1 or sn-3 position.

### 2.6 Gene expression analysis

For each mouse, about 50 mg of each tissue was used for RNA extraction using the RNeasy Tissue Mini Kit (Qiagen, Hilden, Germany) following the manufacturer’s instructions and eluted in 30 μl of UltraPure Distilled Water (#10977035, Invitrogen, CA, USA). Tissues were processed randomly and blindly. The concentration and purity of RNA were determined by measuring the absorbance at 260 and 280 nm, and RNA integrity was assessed by an Agilent2100 Bioanalyzer using the Agilent RNA6000 Nano Kit (#5067-1511, Agilent Technologies, CA, USA). One microgram of total RNA was reverse transcribed using a High-Capacity cDNA Reverse Transcription Kit (#4368814, Applied Biosystems, CA, USA) in a reaction volume of 20 μl.

Sixty-five nanograms total of starting RNA were used to evaluate the expression of the 52 eCBome-related genes and four housekeeping genes (**Supplementary Table 2**; includes information on the function of the genes under investigation) using a custom-designed qPCR-based TaqMan Open Array on a QuantStudio 12K Flex Real-Time PCR System (Thermo Fisher Scientific, CA, USA) following the manufacturer’s instruction as described previously (38). Twenty-four samples (six per group) were analyzed randomly. Gene expression levels were evaluated by the 2^ΔΔCt^ method and represented as fold increase with respect to baseline (LFLS group) within each tissue.

### 2.7 Microbiome analysis

Mouse feces were collected by placing individual mice into an empty sterile cage and allowing them to defecate naturally; feces were collected within 30-min,snap frozen on dry ice and then stored at −80°C until processed. DNA was extracted from feces from *n* = 7–12 mice per diet and time point using the QIAmp PowerFecal DNA kit (Qiagen, Hilden, Germany) according to the manufacturers’ instructions. The DNA concentrations of the extracts were measured fluorometrically with the Quant-iT PicoGreen dsDNA Kit (Thermo Fisher Scientific, MA, USA), and the DNAs were stored at −20°C until 16*S* rDNA library preparation. Briefly, 1 ng of DNA was used as template, and the V2–V3 region of the 16*S* rRNA gene was amplified by polymerase chain reaction (PCR) using the QIAseq 16*S* Region Panel protocol in conjunction with the QIAseq16S/ITS 384-Index I (sets A, B, C, and D) kit (Qiagen, Hilden, Germany). The 16*S* metagenomic libraries were eluted in 30 µl of nuclease-free water, and 1 µl was qualified with a Bioanalyser DNA 1000 Chip (Agilent, CA, USA) to verify the amplicon size (expected size ~600 bp) and quantified with a Qubit (Thermo Fisher Scientific, MA, USA). Libraries were then normalized and pooled to 2 nM and denatured and diluted to a final concentration of 6 pM. Sequencing (2 × 300 bp-paired end) was performed using the MiSeq Reagent KitV3 (600 cycles) on an Illumina MiSeq System (Illumina, CA, USA). Sequencing reads were generated in less than 65 h. Image analysis and base calling were carried out directly on the MiSeq. Data were processed using the DADA2 pipeline (48), and taxonomic assignation was done against the Silva v132 reference database. The operational taxonomic units that were present in fewer than three samples were filtered out, and bacterial abundances were normalized using the cumulative sum scaling (CSS, MetagenomeSeq R package) (49). Microbiota composition was assessed by calculating alpha and beta-diversity indexes obtained using the *Phyloseq* R package, and intra- and inter-individual variations in microbial composition using PERMANOVA (vegan R package) (50). Differential abundance testing of individual taxa was performed using two-way analysis of variance (ANOVA) followed by Tukey’s HSD *post-hoc* test to generate *p*-values.

### 2.8 Statistical analysis

All the statistical analyses (except for microbiome analysis; see above) were performed with GraphPad Prism (version 8.0.1), assessing normality with the Shapiro–Wilk test and identifying outliers using the ROUT test before going on to perform either ANOVAs followed by Tukey’s multiple comparisons tests or Kruskal–Wallis tests followed by Dunn’s multiple comparison tests.

## 3 Results

### 3.1 The effect of whole hemp seed and linseed substitution on anthropometric and metabolic parameters affected by HFHS diet-induced obesity

**Figure 1:**
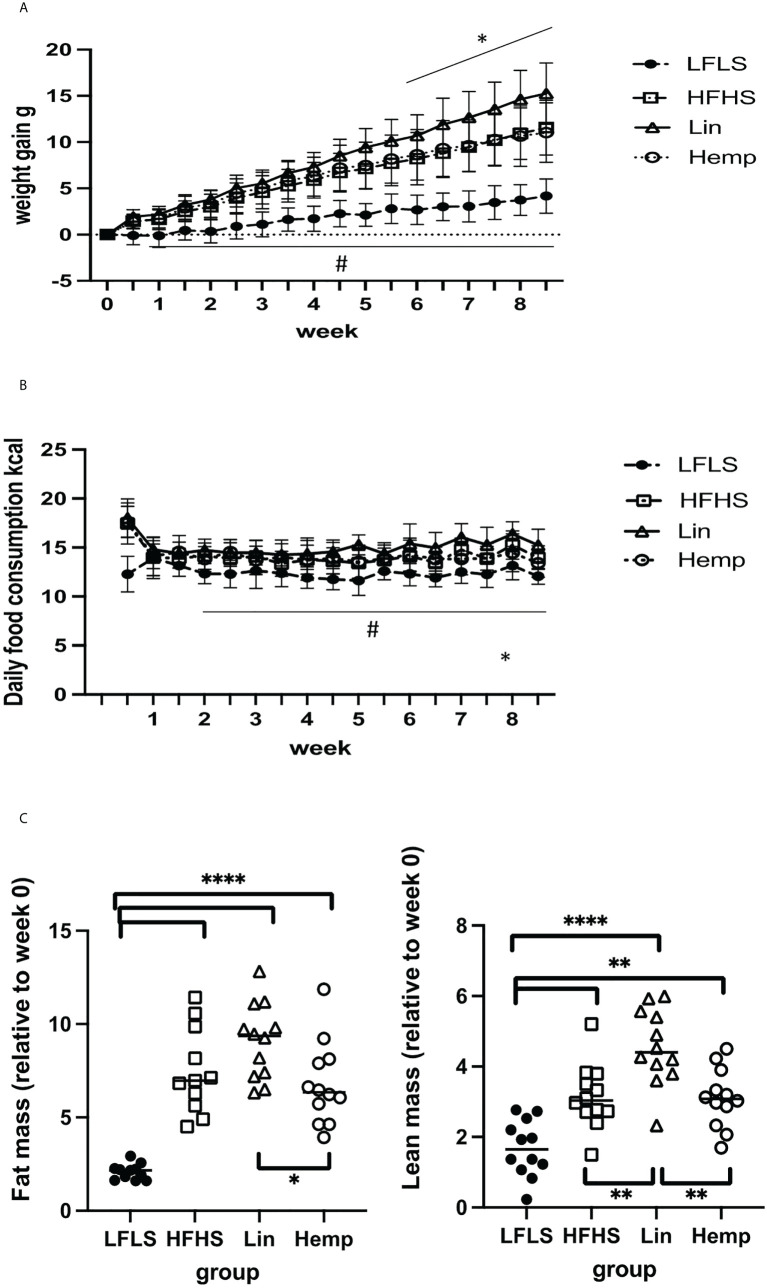
Diet-induced changes in weight gain and body composition. **(A)** Weight gain in g: ^#^significant difference between LFLS versus all the groups fed with HFHS diet (*p* < 0.05); *significant difference between Lin group versus Hemp and HFHS groups (*p* < 0.05). **(B)** Daily food consumption in Kcal: ^#^significant difference between LFLS versus all the groups fed with HFHS diet (*p* < 0.05). *significant difference between Lin group versus Hemp group (*p* < 0.05). **(C)** Body composition changes (difference between week 0 and week 8 in g). *p < 0.05; **p < 0.01; ****p < 0.00001.

To investigate the effects of whole hemp seed dietary substitution on diet-induced obesity and the gut microbiome-eCBome axis, we set up an 8-week protocol in which animals were fed either an LFLS diet, HFHS diet, or an HFHS diet supplemented with either linseeds (Lin) or hemp seeds (Hemp) (**Supplementary Figure 1**). Mice were distributed such that there were no significant differences between the groups in fat mass, lean mass, and fluid mass before the mice were transferred to experimental diets (**Supplementary Figure 3A**). After switching the mice to the experimental diets, all groups fed the HFHS diets gained significantly more weight compared with LFLS group after 1 week and, from week 6, the Lin group started to gain significantly more weight than Hemp and HFHS groups (**Figure 1A**). To investigate whether the differences in weight gain were a result of differences in food intake between groups, we monitored this parameter twice a week. From week 2, the LFLS group consumed significantly fewer calories than all the HFHS groups. At week 8, the Hemp group started to consume significantly less energy than the Lin group (**Figure 1B**). After 8 weeks, we reassessed the fat and lean masses of the mice relative to the start of the diet; all of the HFHS groups had significantly increased fat mass as compared with the LFLS group; however, interestingly, the Hemp group had significantly less fat than the Lin group (**Figure 1C**). Lean mass was also increased in all HFHS groups, with the Lin group having significantly more lean mass than all other groups (**Figure 1C**). When correcting the fat or the lean mass by the total weight of the mice, we observed increases in all HFHS groups as compared the LFLS controls, but no differences between the HFHS groups were identified (**Supplementary Figure 3B**).

**Figure 2:**
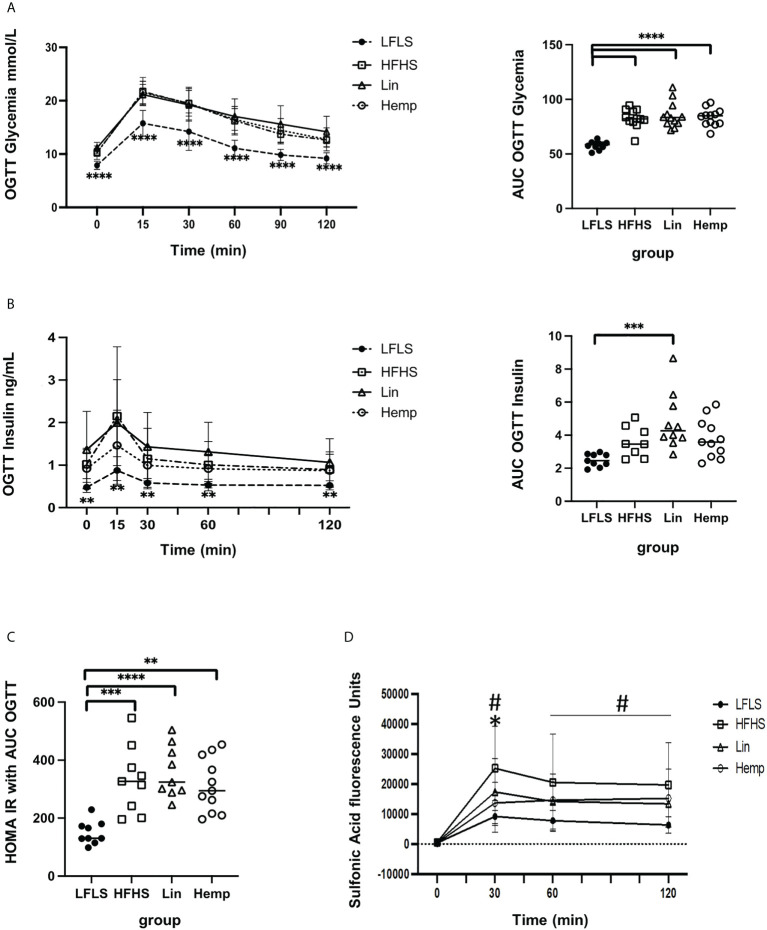
Glucose and insulin homeostasis after 8 weeks of HFHS diets. **(A)** Glycemic excursion curves during OGTT (left, significant difference between LFLS vs. all the groups fed with HFHS diets) with the corresponding area under the curve analysis of glucose (right); **(B)** Insulinemic excursion curves during OGTT (left, significant difference between LFLS vs. all the groups fed with HFHS diets) and the corresponding area under the curve analysis; **(C)** HOMA-IR calculated using glucose and insulin AUCs (area under the curve) from OGTTs. *p < 0.05); **p < 0.01; ***p < 0.001); ****p < 0.00001). **(D)** Intestinal permeability as measure by sulfonic acid levels in blood after administration with the OGTT: ^#^significant difference between LFLS versus HFHS group (*p* < 0.05). *significant difference between Hemp versus HFHS group (*p* < 0.05).

We then assessed glucose handling within the mice by administering an OGTT. The LFLS group had lower blood glucose levels at each time point measured as compared to the HFHS groups, with no differences observed between the latter, and area under the curve analysis showed the same pattern (**Figure 2A**). The measurement of insulin levels during the OGTT time course mirrored the results obtained for glucose levels; however, when we calculated the areas under the curves, only the Lin group was found to be significantly higher than the LFLS group (**Figure 2B**). The calculation of a HOMA-IR from the above curves found that all the HFHS groups had increased values with respect to the LFLS group (**Figure 2C**).

In order to determine whether the diets affected intestinal permeability, we co-administered sulfonic acid during the OGTT. The LFLS group exhibited the lowest intestinal permeability as determined by the level of sulfonic acid within the blood at each time point. At the 30-min peak of sulfonic acid levels, however, we detected significantly lower levels in the Hemp group versus the HFHS group (**Figure 2D**), suggesting that hemp seed substitution induced an improvement in intestinal permeability.

**Figure 3:**
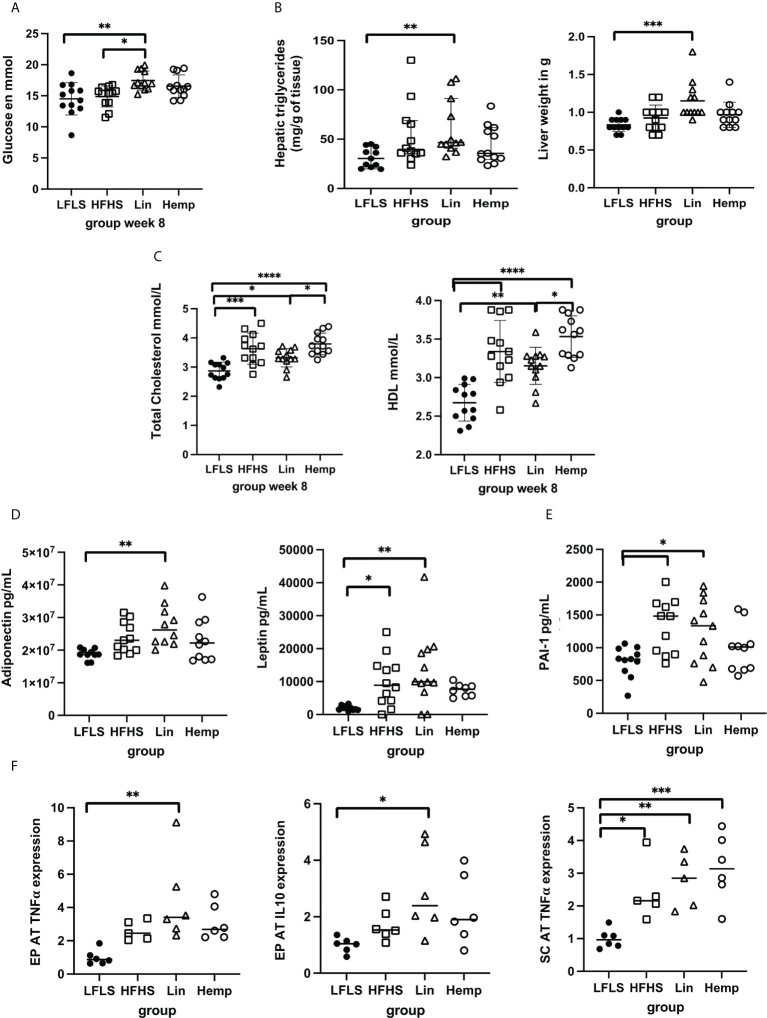
Assessment of metabolic parameters, cytokines, and adipokines after 8 weeks of HFHS diets. **(A)** Fasting glucose levels; **(B)** liver triglyceride content and weight; **(C)** total cholesterol and HDL levels; **(D)** circulating levels of adipokines; **(E)** circulating levels of PAI-1; **(F)** epididymal (EPAT) and sub-cutaneous (SCAT) levels of TNF-α and IL-10. *p < 0.05; **p < 0.01; ***p < 0.001; ****p < 0.00001.

We also measured the effects of the different diets on several biochemical parameters in mice that were fasted for 12 h before sacrifice. In line with the results from the OGTT demonstrated above, the Lin group had higher fasting glucose levels than both the LFLS and the HFHS groups, whereas the HFHS and Hemp groups showed no such increase (**Figure 3A**). No significant differences in plasma triglyceride levels were observed among the groups (data not shown); however, triglyceride levels were significantly increased only within the livers of the Lin group compared with the LFLS group (**Figure 3B**). This coincided with the Lin group also having significantly heavier livers than the LFLS group (**Figure 3B**). All of the HFHS groups had significantly higher total cholesterol levels than the LFLS group, with the Lin group having lower total cholesterol than the Hemp group (**Figure 3C**). This was apparently contributed to by lower HDL cholesterol within the Lin versus the Hemp group (**Figure 3C**); however, the HDL/total cholesterol ratio was not different between groups (data not shown).

### 3.2 The effect of whole hemp seed and linseed substitution on hormones, inflammatory cytokines, and markers of fat browning and lipid metabolism

Within 12-h fasted animals, we did not detect any significant differences between the groups in basal ghrelin, GIP, GLP-1, or insulin plasma levels (data not shown). Adiponectin levels where highest in the Lin group, being the only group to have a statistically significant increase over the LFLS group (**Figure 3D**), whereas resistin levels were increased in all the HFHS groups compared with the LFLS group (data not shown). Leptin levels were significantly higher in the HFHS and Lin groups versus LFLS, but not in the Hemp group, compared with the LFLS group (**Figure 3D**). Despite this, there were no differences between the levels of any of these factors between the different HFHS groups.

We also assessed different inflammatory mediators within the plasma of mice. TNF-α, IFN-γ, IL-17, IL-10, IL-6, and the IL-1β levels were not significantly different among groups (data not shown). Interestingly, however, PAI-1 levels were significantly increased in the Lin and HFHS groups versus the LFLS group, but not in the Hemp group (**Figure 3E**). When we went on to look at the gene expression if these cytokines in adipose tissue depots, we found that, in epididymal adipose tissue (EPAT), *IL-10 and TNF-*α gene expression were only increased in the Lin versus the LFLS group (**Figure 3F**), whereas no differences were found in *PAI-1* or *IL-6* expression among the groups (data not shown). In subcutaneous adipose tissue (SCAT), *TNF-*α expression was increased in all the HFHS groups versus LFLS, whereas no differences were found in *IL-10*, *IL-6*, and *PAI-1* gene expression among the groups (**Figure 3F** and data not shown).

We also evaluated the gene expression of browning markers in various adipose tissue depots. No differences in *Pparg1a*, *Prdm16*, or *Ucp1* expression were observed in brown adipose tissue (BAT) between the different groups (data not shown). In the EPAT, *Cidea* expression had decreased expressed in all the HFHS versus the LFLS groups, and *Pparg1a was* decreased in the Lin versus the LFLS groups (**Supplementary Figure 4**), but no differences in *Prdm16* and *Ucp1* were observed among the different groups (data not shown). In the SCAT, *Cidea* and *Pparg1a* expression was decreased in in all the HFHS groups versus the LFLS group (**Supplementary Figure 4**), whereas, again, no differences were found in *Prdm16* and *Ucp1* expression (data not shown). Evaluating the gene expression of lipid metabolism enzymes in the EPAT and the SCAT revealed that *Acc1*, *Atgl, Hsl*, and *Scd1* were all downregulated in the HFHS versus the LFLS groups (**Supplementary Figure 5**).

### 3.3 eCBome gene expression and mediator-level results

To assess the direct effects of the different diets on the eCBome, we measured eCBome gene expression and mediator levels in the muscle, hypothalamus, liver, BAT, SCAT, and EPAT, because it is well established that endocannabinoids and related lipids play important roles in each of these tissues with respect to the regulation of various aspects of metabolism (7, 10, 12, 16, 18, 20, 25) and plasma (lipid levels only). As expected, we observed important changes in eCBome signaling with the hemp seed and linseed supplemented diets, which are rich in PUFAs.

#### 3.3.1 Genes encoding eCBome receptors

**Figure 4:**
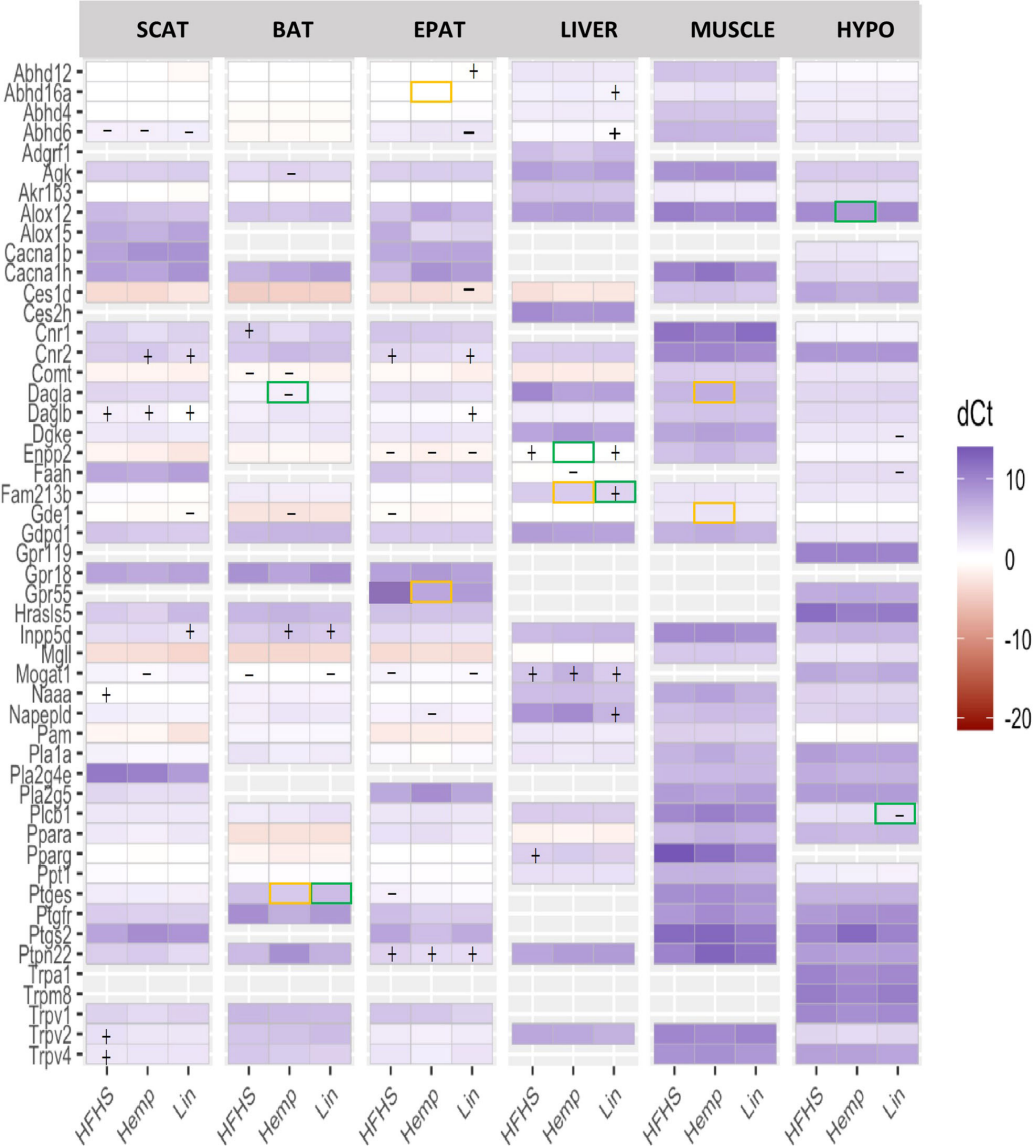
eCBome receptor and enzyme expression measured by qPCR array in brown adipose tissue (BAT), epididymal adipose tissue (EPAT), subcutaneous adipose tissue (SCAT), hypothalamus (HYPO), liver, and muscle. Statistically significant differences between the high-fat high sucrose groups (HFHS, Hemp, or Lin) *versus* the LFLS group are indicated with a “+” (increase in expression) or “−” (decrease in expression); *p* < 0.05. Yellow boxes indicate significant difference between Hemp and Lin; green boxes indicate significant differences between Hemp or Lin and HFHS; *p* < 0.05.

Receptor gene expression and response to the diets was different from one tissue to another. Within the BAT, we observed changes in the expression of *Cnr1* and *Cacna1h*, where *Cnr1* (encoding CB1) was more highly expressed in all the HFHS group compared with the LFLS group, but this increase was rendered non-significant in the Hemp and Lin groups. In the EPAT and the SCAT, we saw changes in the expression of *Cnr2* (encoding CB2), which was more strongly expressed in the HFHS and Lin versus the LFLS group in the EPAT and more strongly expressed in the Hemp and Lin versus the LFLS group in the SCAT. In the SCAT *Trpv2* and *Trpv4*, gene expression was increased in the HFHS group compared with the LFLS group but not in the Hemp or Lin groups. In the liver, *Pparg* expression was increased in the HFHS group versus the LFLS group and, again, the addition of hemp seed or linseed rendered the difference insignificant. No significant differences were found in the muscle and hypothalamus (**Figure 4**).

#### *3.3.2 N*-acylethanolamine anabolic enzymes

Regarding the *N*-acylethanolamine (NAE) anabolic enzymes, differences were also observed among the various tissues and, here, we focus on those changes that might explain the observed changes in mediator levels. For the BAT, we observed that *Gde1* expression was decreased in the Hemp group compared with LFLS, whereas *Inpp5d* expression increased in the Hemp and Lin groups versus LFLS. Most changes were observed in the EPAT, where the expression of five enzymes was altered, with *Gde1* and *Ptges* being less expressed in all HFHS groups compared with LFLS group and *Napepld* being decreased in the Hemp versus LFLS group. Conversely, *Ptpn22* expression was increased in all the HFHS groups compared with LFLS group. *Abhd4* expression decreased in the Lin compared with Hemp group (**Figure 4**). In the SCAT, changes were observed for the expression of two enzymes only: *Gde1* expression decreased, whereas *Inpp5d* expression increased in the Lin compared with LFLS group. For the liver, both *Fam213b* and *Napepld* expression increased in the Lin group, being higher compared with all the other groups for *Fam213b*, but only versus the LFLS group for *Napepld*. Finally, in the muscle, *Gde1* expression was increased in the Lin compared with the Hemp group (**Figure 4**).

#### 3.3.3 *N*-acylethanolamine catabolic enzymes

For the BAT, *Ptges* expression was lower in the HFHS and Hemp groups versus Lin. In EPAT, *Ptges* was less expressed in the HFHS group compared with LFLS group. Concerning the SCAT, *Naaa* expression was increased in the HFHS versus LFLS group, an effect that as not observed in Hemp or Lin. The liver had decreased *Faah* expression only in the Hemp versus the LFLS group. Within the hypothalamus, *Faah* expression was decreased in the Lin versus LFLS group (**Figure 4**).

#### 3.3.4 2-Monoacylglycerol anabolic enzymes

For the BAT, *Dagla* expression was decreased in the Hemp group versus HFHS and LFLS groups. In the EPAT, the changes were mostly found in the HFHS groups, where *Daglb* was more expressed in the Lin versus the LFLS group. Concerning the SCAT, *Daglb* expression was increased in all the HFHS groups compared with LFLS. For the hypothalamus, *Dgke* was decreased in the Lin group versus the LFLS and *Plcb1* was decreased in Lin versus HFHS and LFLS. Finally, in the muscle, *Dagla* was more expressed in the Lin versus the Hemp group (**Figure 4**).

#### 3.3.5 2-Monoacylglycerol catabolic enzymes

In the BAT, *Mogat1* was less expressed in HFHS and Lin group versus LFLS. In the EPAT, *Enpp2* and *Mogat1* were decreased in the HFHS groups versus the LFLS group. *Abhd6* and *Ces1d* expression was decreased, whereas *Abhd12* expression was increased, in the Lin compared with the LFLS group. Concerning the SCAT, *Abhd6* expression was lower in all the HFHS groups compared with LFLS, whereas *Mogat1* expression was decreased in Hemp. In the liver, *Enpp2* increased in the HFHS and Lin groups versus the LFLS but not in the Hemp, in which expression was significantly lower than in HFHS. *Abhd16a* and *Abhd6* expression was higher only in the Lin than the LFLS group. *Mogat1* expression was stronger in all the HFHS groups than the LFLS group. In the hypothalamus, *Alox12* expression was increased in the Hemp compared with the HFHS group (**Figure 4**).

Together, the above data emphasize how different eCBome anabolic and catabolic enzyme expression may contribute to alterations in both omega-3 PUFA- and omega-6 PUFA-derived mediator levels within in a tissue- and treatment-dependent manner.

#### 3.3.6 eCBome mediators

eCBome mediator levels were measured in the BAT, EPAT, SCAT, liver, hypothalamus, muscle, and plasma. As expected, important changes in eCBome mediator concentrations under the Hemp and Linseeds supplemented diets, which are rich in PUFAs.

The assessment of FA levels showed an increase in omega-3 and a decrease in the omega-6 FAs in several tissues of the mice fed the supplemented diets. In the adipose tissues (BAT, EPAT, and SCAT), the liver, the muscle, and the hypothalamus the omega-3 FA SDA and at least one other omega-3 FA (EPA, DPA and/or DHA) increased in the Hemp and the Lin versus the LFLS group and, in fact, were almost always increased in comparison to the HFHS group (**Supplementary Figure 6**). These changes were not observed in plasma, where instead DHA was found to be decreased in the Hemp and Lin groups compared with HFHS. While the omega-6 FAs assessed (AA and LA) were less affected, they were decreased in the Lin group, in the BAT and muscle, compared with the LFLS group.

**Figure 5:**
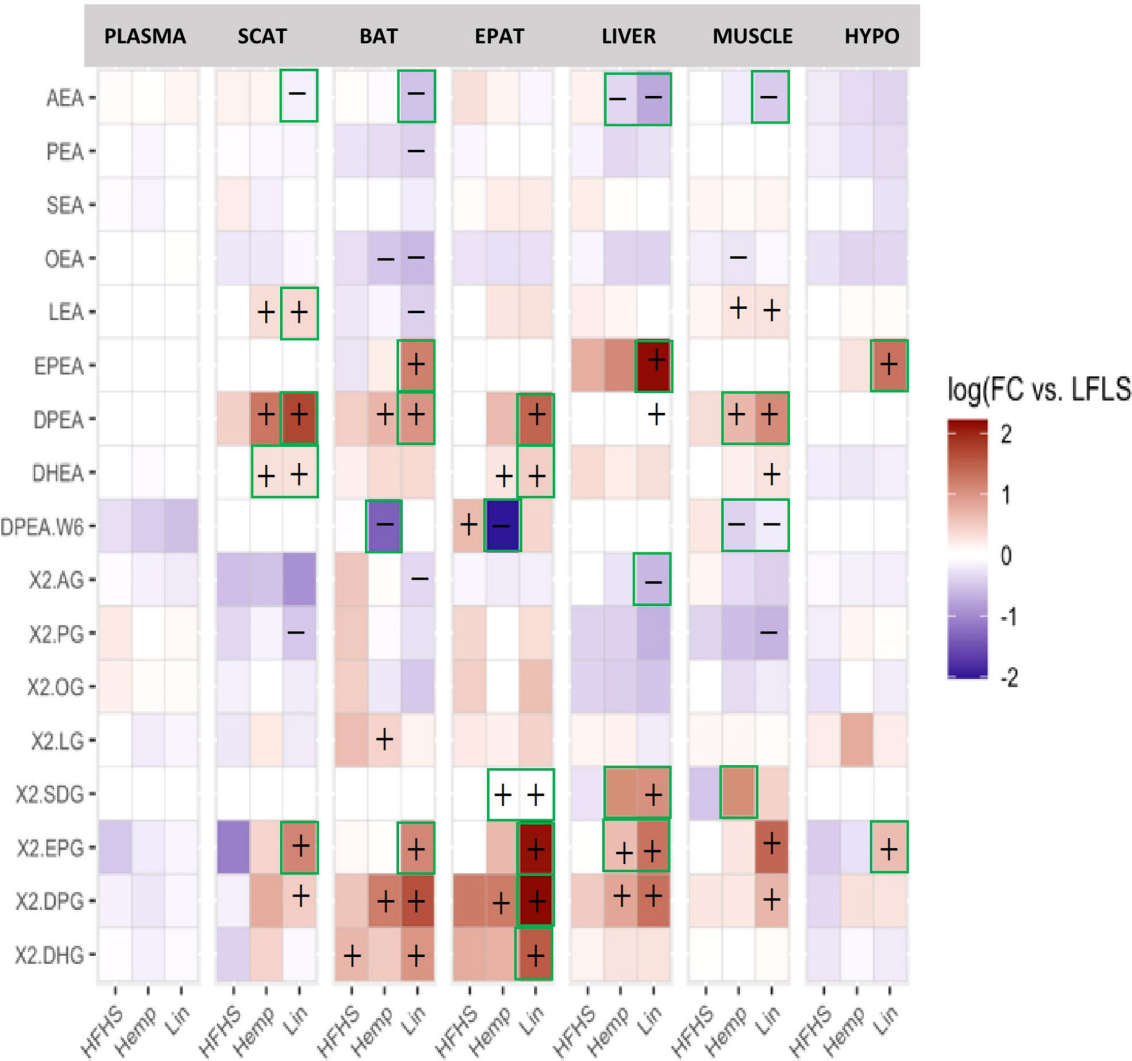
eCBome bioactive lipid mediator levels in plasma, brown adipose tissue (BAT), epididymal adipose tissue (EPAT), subcutaneous adipose tissue (SCAT), hypothalamus (HYPO), liver, and muscle. Statistically significant differences between the high-fat high sucrose groups (HFHS, Hemp, or Lin) *versus* the LFLS group are indicated with a “+” (increase in expression) or “−” (decrease in expression); *p* < 0.05. Green boxes indicate significant differences between Hemp or Lin and HFHS; *p* < 0.05.

Likewise, at least two of the omega-3 FA-containing NAEs (EPEA, DPEA, and DHEA) increased in the Hemp and Lin groups in almost all the tissues; whereas the omega-6 derived NAE, AEA, decreased in all the tissues (except the EPAT and hypothalamus) of the Lin group (**Figure 5**). AEA was also decreased by hemp seed substitution in the liver (where it is known to be a driver of hepatosteatosis), both with respect to LFLS and HFHS. The omega-6 DPA-containing DPEA decreased in the Hemp group in the BAT, EPAT, and the muscle (where linseed substitution decreased it as well.

For the 2-MAGs (**Figure 5**), once again, at least one of the omega-3 FA containing 2-MAGs (2-SDG, 2-EPG, 2-DPG, and/or 2-DHG) increased in the Hemp and Lin groups in all the tissues, whereas the omega-6-FA-containing 2-AG decreased in the Lin group in the BAT and liver.

Taken together, these data indicate that eCBome mediator levels are sensitive to even a relatively low level of dietary substitution with either hemp seed or linseed, effectively increasing the levels of omega-3 FA-derived eCBome lipid mediators. In particular, SDA and to a less broad extent 2-SDG, levels were remarkably higher in tissues of the Hemp group (**Figure 5**; **Supplementary Figures 6, 7**).

### 3.4 Hemp seed and linseed substitution effects on gut microbiota

We next assessed the gut microbiota of feces from the mice on first on the chow diet and then in response to the dietary changes. Metataxonomic profiling showed that the overall gut microbiota architecture was different between the period on which they were fed the chow diet (week 0) and after LFLS, HFHS, Hemp, and Lin diets (week 8); however, Shannon alpha diversity and *Firmicutes: Bacteroidetes* phylum ratios were not found to be affected (**Supplementary Figure 8**). Differential abundance testing of family taxa identified *Akkermansiaceae*, *Bifidobacteriaceae*, *Clostridiaceae 1*, *Eggerthellaceae*, *Enterococcaceae*, *Peptostreptococcaceae*, *Pseudomonadaceae*, *Staphylococcaceae*, and *Streptococcaceae* abundance to have been increased in one or more of the diets after 8 weeks of being taken off chow (i.e., in the LFLS, HFHS, Hemp, and Lin groups at week 8 as compared with week 0), whereas the *Anaeroplasmataceae* and *Lactobacillaceae* family abundances decreased (**Supplementary Figure 9**). We then attempted to identify the differences at the family level after 8 weeks of the experimental diets. Differential abundance testing identified changes in only two families: *Clostridiaceae 1*, which was more abundant in Hemp versus Lin, and *Rikenellaceae* was more abundant in the Hemp and HFHS versus the LFLS group (**Supplementary Figure 10**).

## 4 Discussion

We postulated that partial substitution of the fat in an HFHS diet with hemp seed, being a rich source of omega-3 FAs, would have benefits on health in part by modulating the eCBome and the gut microbiome. We found that, toward the end of the study, the Lin group gained significantly more weight than the HFHS group, whereas hemp seed substitution had no effect on body weight. This difference may be due to the fact that the Lin group tended to consume more calories than the HFHS and Hemp group and, at the end of the study, consumed significantly more calories than the Hemp group. Concomitantly, the Lin group had the highest fat mass of all the groups and significantly more fat than the Hemp group. Accordingly, leptin levels, which are a reflection of the amount of fat mass (51), were significantly higher in Lin group compared with the LFLS group, whereas the Hemp group showed no difference. Similarly, adipose-derived adiponectin plasma levels were significantly elevated in the Lin group but not in the HFHS or Hemp groups. These results are consistent with the observation that linseed substitution increased weight gain in our mice and suggest that, at least within our experimental conditions, this dietary regimen exacerbates weight gain. This increase in weight gain makes it difficult to assess the effects of our linseed diet on the subsequent metabolic parameters assessed and discussed below, as it is not possible to distinguish effects observed in the Lin group as being the result of the linseeds themselves, or simply the increased weigh gain that they induced. Interestingly, Shafie et al. recently showed that whole linseed substitution increased weight gain in rats in a corn starch diet and tended to do the same in a high carbohydrate, high-fat diet (44).

In conjunction with the Lin group having the highest weight, glycemia was also highest in the Lin group, whereas the HFSH and Hemp groups did not differ from the LFLS control. Consequently, the AUC of insulin levels during the OGTT was significantly higher only in the Lin versus the LFLS group. Despite this, all HFHS groups had similar glucose levels during the OGTT, together suggesting that the Lin group had a higher level of insulin resistance as compared with the HFHS and Hemp groups and was likely approaching a diabetic state, whereas the other HFHS groups were likely prediabetic. Accordingly, the HOMA IR index, which is positively correlated with insulin resistance (52), was higher in all HFHS groups, with the HFHS and Lin groups having the highest level of statistical significance as compared with the LFLS group. Thus, in accordance with it having significantly less fat than the Lin group, the Hemp group appeared to have less severe insulin resistance compared with the Lin group. Furthermore, the Lin group was also the only one showing increased levels of liver TGs and weight with respect to the LFLS group, which may be related to the above mentioned worsening of glucose handling given the link between liver fat levels and insulin resistance (53).

Despite hemp seed substitution having no effect on weight gain or adiposity as compared to the HFHS group it decreased sulfonic acid uptake, indicating that it preserved intestinal barrier integrity. Intestinal barrier breakdown is a feature of obesity and contributes to increased adipose tissue inflammation and circulating inflammatory cytokine levels (54). Interestingly, the assessment of circulating inflammatory parameters showed that PAI-1, which is upregulated by pro-inflammatory cytokines such as TNF-α (55) and increased in obesity and in diabetes and is involved in thrombosis, atherosclerosis as well as ischemic cardiovascular events (56, 57), was significantly higher in the Lin and HFHS groups, but not in the Hemp group, versus the LFLS group. Thus, it appears that hemp seed substitution mitigated the HFHS-induced increase in PAI-1 levels, even though it only showed a trend toward a decrease as compared to the HFHS group. This may have been due to the fact that the study was under-powered to allow for the detection of a statistically significant decrease. Regardless, PAI-1 augments mucosal damage (58), thus it is possible that hemp seed substitution, by rendering the HFHS-mediated increase in PAI-1 non-significant, may preserve mucosal integrity, which would be interesting to investigate in future studies.

In line with the increased adiposity observed in the Lin group, in the EPAT, *IL10*, which is linked to insulin resistance in the adipose tissue (59), and *TNF-*α, a pro-inflammatory cytokine that plays a role in the pathophysiology of insulin resistance (60), were increased in the Lin, but not Hemp, group versus the LFLS group. It should be noted however, that these results are driven by relatively few animals within the Lin group, and thus should be interpreted with caution; regardless, they are consistent with the data presented above on the effects of linseeds on weight gain in mice on an HFHS, suggesting that whole linseed substitution is pro-obesogenic, resulting in increased weight gain and expression of inflammatory markers associated with excessive fat accumulation, while hemp seeds do not produce this effect. However, the link between increased intestinal barrier integrity and decreased circulating PAI-1 levels in the hemp seed group does not appear to be linked to modification of *Tnf-*α expression within adipose tissue depots. The low efficacy of linseed substitution against the effects of a high-fat diet was reported also by Shafie et al. (44), who showed that whole linseeds are less effective than linseed components at reversing diet-induced metabolic syndrome in rats. On the other hand, the protective results observed in the Hemp group are partially in agreement with those reported by Opyd et al. (61), who showed that hemp seeds were more effective than hemp oil at regulating lipid metabolism in obese Zucker rats. We, however, were unable to detect any beneficial effects in either the Lin or Hemp groups on total cholesterol levels in mice, which may be due to species-specific differences or the use of our diet-induced obesity model as compared with a genetic model. That being said, it was interesting to note that while the Lin group had significantly lower total cholesterol levels as compared with the Hemp group, this difference appears to be driven by “healthy” HDL cholesterol in the Lin group, lower levels of which are associated with increased risk of the development of metabolic syndrome including increased fasting glucose levels (62).

It is important to note that all the HFHS diets were isocaloric with very similar energetic proportions from protein (20%), carbohydrates (35%), and fat (45%) from fat and that they had similar fiber content (**Supplementary Figure 2**). However, we cannot exclude the possibility that the diets differed significantly in the quantity and quality of macro and micronutrients, such as minerals, vitamins, polyphenols etc. which may have affected our results. As mentioned above, the substituted amount of seeds was established on the one hand, on the basis that this amount of linseeds was shown to be sufficient to result in effects in mice (21), and on the other hand, on the idea that a clinical study could be performed with 15% of dietary fat coming from seeds, because this represents about 37 g of whole hemp seeds that can be easily consumed daily. While the seed substitution resulted in demonstrable changes, many statistically insignificant trends were observed, which may have translated to significant results should a less severe HFHS diet has been used, or had we extended the study beyond 8 weeks, because, for example, the Lin group only became heavier than the HFHS group in the last 2 weeks.

As expected, important changes in eCBome signaling under the hemp and linseed substituted diets, which are rich in PUFAs, were found in the present study. The ECS generally decreases energy expenditure and increases energy storage in several organs through the elevation of AEA and/or 2-AG levels and CB1 receptor activation (10). However, the ECS is now considered as part of a much larger signaling system, the eCBome, which, with its over 100 lipid mediators and about 13 molecular targets (including the PPARs, thermosensitive TRP channels such as TRPV1, and orphan GPCRs such as GPR119), is also deeply involved in energy metabolism control but often in ways opposite to endocannabinoid/CB1 signaling (63). Mediators whose ultimate precursors are omega-3 FAs such as the EPEA and DHEA (24), have targets that are being actively investigated in the framework of inflammation (11). In fact, there is a competition between the omega-6 and omega-3 FA precursors in the production of the corresponding eCBome mediators, such as NAEs and 2-MAGs, because all members of these two families of lipids share the same anabolic enzymes (14). As a consequence, the higher the intake of omega-6 FAs and their precursors such as LA, the more such FAs will be esterified into phospholipids wherefrom NAEs and 2-MAGs are biosynthesized, and the same applies to dietary omega-3 FAs and their precursors such as ALA (64). In line with this, our results show a general increase in the omega-3 FAs and a decrease in the omega-6 FAs, and similar changes in their respective NAEs and 2-MAGs, in several tissues coming from mice fed the diets substituted with Lin or Hemp seeds. In particular, the omega-3 FAs, SDA, EPA, DPA, and DHA increased in the Hemp and the Lin groups versus the LFLS group, whereas the omega-6 FAs AA and LA decreased in almost all the tissues of mice belonging to the Lin group. Likewise, the omega-3–derived NAEs, EPEA, DPEA, and DHEA increased in Hemp and the Lin groups, whereas the omega-6 NAEs, AEA, and DPEA decreased in either only the Lin or both the Hemp and Lin groups, respectively. Concerning the 2-MAG results, once again, the omega-3–derived 2-SDG, 2-EPG, 2-DPG, and 2-DHG increased in both the Hemp and Lin groups, whereas the omega-6 2-AG decreased in the Lin group. Regarding our above considerations on omega-3 and omega-6FAs, it must be emphasized that we did not measure here the levels of such FAs esterified to phospholipids, which act as eCBome mediator biosynthetic precursors, but only the free FAs, which may also reflect the enzymatic hydrolysis of the corresponding eCBome mediators. Regardless, our study shows just how sensitive the eCBome is to relatively minor changes in dietary fat intake, given that only 15% of the fat in the HFHS diet was replaced by hemp or linseed-derived fat. This level of substitution is roughly equivalent to an average sized human consuming only 2 tablespoons of seeds a day approximately, suggesting that it can similarly affect eCBome levels in humans, as shown in studies usually using fish- and krill-derived FAs (22, 65).

Indeed, although the alterations in the tissue levels of eCBome mediators observed in our study can be explained mostly by the effects of the substituted diets on the levels of the ultimate precursors of such mediators, changes in the expression of eCBome anabolic and catabolic enzymes may also contribute to such alterations. The hemp seed diet downregulated the expression of *Napepld* (a main anabolic enzyme for NAEs) mRNA levels versus the LFLS diet in the EPAT, and *Dagla* (a major anabolic enzyme for 2-MAGs) mRNA levels versus the HFHS and LFLS diets in the BAT, and versus the Lin diet in the muscle; both genes encode for ECS anabolic enzymes. Conversely, the linseed diet generally increased anabolic enzyme and decreased catabolic enzyme expression. In the liver, *Napepld* mRNA levels increased in the Lin versus the LFLS group and, in the EPAT, *Daglb* (a main enzyme, which biosynthesizes 2-MAGs) mRNA levels were higher in Lin versus the LFLS group. Moreover, in the hypothalamus, FAAH (which degrades NAEs and to a lesser extent 2-MAGs) expression was decreased in the Lin versus LFLS group. These data suggest that the contribution of metabolic enzyme expression to the diet-induced alterations in NAE and 2-MAG levels observed here might be different depending on the administered diet, with anabolic enzymes contributing to either omega-6– or omega-3–derived mediator levels during hemp seed or linseed substitution. Given the opposite changes often observed in omega-3 versus omega-6 PUFA-derived NAEs, it would be interesting to hypothesize that NAE biosynthetic enzymes contribute less than availability of NAE ultimate precursors to NAE tissue concentrations, and that different anabolic enzymes may contribute to omega-3 (i.e., *Gde1*, *Napepld*, and *Abdh4*, but not in the liver or muscle) versus omega-6 PUFA-derived (i.e., *Inpp5d*) NAEs. While with respect to 2-MAGs, in some tissues (EPAT, muscle), *Dagla* or *Daglb* may contribute to increased levels of omega-3 PUFA-derived 2-MAGs in the treated groups, and *Daglb* to increase MAGs in the SCAT following HFHS diets. These intriguing hypotheses will need to be investigated in future studies.

Interestingly, SDA and the corresponding MAGs, 1 (3)- or 2-SDG, increased significantly in the Hemp and Lin groups in the different tissues, and especially in the adipose tissue. SDA is a rare omega-3 FA in nature and mainly contained in Hemp (66, 67) and other seeds (68). Several clinical and fundamental studies reported the beneficial effect of SDA substitution in improving lipid profiles such as dyslipidemia (41, 42), atherosclerosis (69), cardiovascular disease (70), hepatic steatosis (71), inflammation (72), and cancer (73). Our results suggest that SDA, and possibly its metabolites, might be partly responsible for the blunting of the increase in PAI-1 levels observed in response to the HFHS by hemp seed substitution, an increase that was unaffected by linseed substitution. While 1/2-SDG levels were similarly affected in the Hemp and Lin groups, except in muscle where Hemp resulted in a significant increase over Lin, differences in other SDA-derived metabolites (i.e., *N*-straeridonoylethanolamine; SDEA) may be present within the mice, which we were not able to measure at the time of this study. However, we have since developed the methodology to quantify this eCBome mediator and have found that murine cells are able to produce SDEA in response to incubation with SDA (data not shown, manuscript in preparation).

A recent meta-analysis on the rate of mortality associated with levels of omega-3 FAs highlighted how mortality for all causes as well as for cardiovascular disease and cancer is inversely correlated to the levels of circulating long chain (20–22 carbon) omega-3 FAs but not to those of ALA (74). These findings are in agreement with the poor conversion of ALA to SDA and, hence, to EPA and DHA because of the lack of an efficacious desaturase enzyme in humans and other mammals (75), and highlight the potential importance of dietary SDA. This omega-3 FA, by bypassing the necessity of such conversion, and being in turn converted to EPA and DHA (partly), might ensure amounts of C20–22 omega-3 FAs (and their metabolites) that are optimal to counteract several chronic societal disorders and, hence, act as a surrogate of dietary EPA and DHA. Future studies will need to address the question of whether also 1- and 2-SDG and SDEA (the latter of which, however, we cannot detect here) play beneficial actions against metabolic disorders, and whether such actions are due to anti-inflammatory effects similar to those reported for DHEA and EPEA.

Concerning the gut microbiota results, the metataxonomic analysis performed here showed that only few differences can be induced by hemp seed or linseed substitution versus the LFLS or HFHS diets. The paucity of the observed changes might be due to the fact that our control (LFLS) diet did not contain considerable amounts of fiber, as in typical chow diets. Thus, the amount of seed may not have been sufficient to overcome the changes induced by the experimental diets over chow. Alternatively, possibly contrasting effects of hemp seed fiber, FA or protein contents may explain the small effect on fecal microbiota composition given that previous studies with linseed oil found that linseed oil substitution within a high-fat diet improved gut microbiota diversity, reducing the abundance of the *Firmicutes* phylum in mice (76). A recent study with hemp seeds found improved gut microbiota profile by decreasing the relative abundance of *E. coli* and increasing that of *Bifidobacterium* and *Lactobacillus* species after 11 weeks of dietary intervention in mice (77). At any rate, we did observe here some differences at the family level, where the *Clostridiaceae 1* was more abundant in Hemp versus Lin group, and *Rikenellaceae* was more abundant in the Hemp and HFHS versus the LFLS group. The interpretation of these results seems difficult due to the paucity of information on the role in metabolism and inflammation of these two families; however, *Clostridiaceae 1* abundance has been found to correlate with increased mucosal thickness in association with decreased inflammation (78), which may be of relevance to the decreased intestinal permeability we observed in mice fed hemp seeds. Furthermore, while contradictory evidence exists with respect to the association of *Clostridiaceae* with obesity, this family has recently been reported to be more abundant in lean mice (79) and humans (80) as well as being associated with decreased type 2 diabetes (81). A previous study in piglets showed that fish oil alleviates inflammation and parenteral nutrition-associated liver diseases and intestinal injury while concomitantly increasing *Rikenellaceae* (82), a family also associated with anti-inflammatory actions against ulcerative colitis (83). It is important to note that the microbiomes of mice and humans are not particularly similar and may very well react overall differently to any given dietary component: This makes generalizing difficult, especially given that few families were identified in our studies has having responded to the diets. Furthermore, our analyses did not identify specific genera that can account for the different abundances of the above families. Regardless, our study supports the notion that whole hemp seeds have potentially beneficial prebiotic characteristics, which may be further enhanced through fermentation (84, 85). The contribution of individual hemp seed components responsible for the above discussed effects remain to be identified in future studies, however, with SDA and SDA-rich oils being of particular interest to metabolic health and effects on the microbiome (manuscript in preparation).

In conclusion, the results of this study suggest that whole linseed substitution of an HFHS diet results in increased weight gain and adiposity, which likely results in the worsening of some metabolic and inflammatory parameters, a worsening that is not observed when using instead hemp seed substitution. Hemp seed substitution of the HFHS, in addition to having no effect on weight or adiposity, instead abrogated HFHS-induced elevation of inflammatory markers in association with improved gut permeability. However, eCBome modulation was similar with the two diets, even though Linseeds contain three times more omega-3 FAs than hemp seeds. In fact, the observed metabolic and inflammatory marker changes were associated with a general decrease in AEA and an increase in omega-3 FA containing endocannabinoidome bioactive lipids. Additionally, more beneficial effects on the gut microbiome by the hemp seed versus linseed substitution may have been partly unnoticed in our study, due to the comparison with a LFLS diet instead of a chow diet, which might also explain some of the differences observed here between the two diets, especially in relation to the inflammatory profile of the adipose tissue. Thus, hemp seed-containing foods might represent a source of healthy fats and effective nutrients that are not likely to exacerbate the metabolic consequences of the commonly consumed, obesogenic diets in Western societies, while producing some anti-inflammatory actions.

## Data availability statement

All 16S sequencing data has been deposited in the NCBI with SRA accession number of PRJNA809548.

## Ethics statement

The animal study was reviewed and approved by Institutional Review Board of the Laval University for the protection of animals (license # 2018101-1).

## Author contributions

Conceptualization, CS; investigation, RB, CMan, SL, CMar and NF; writing, RB, CS and VD review and editing, NF, CS and VD visualization, RB, SL and CS; supervision, CS and VD project administration, RB; funding acquisition, CS and VD. All authors have read and agreed to the published version of the manuscript.

## Funding

RN was supported by a Mitacs grant (IT15195) obtained in partnership with Nature’s Decision, who also provided the hemp seeds *via* Igor Kovalchuk (University of Lethbridge, Canada). VD is the holder of the Canada Excellence Research Chair on the Microbiome-Endocannabinoidome Axis in Metabolic Health (CERC-MEND) at Université Laval, funded by the Federal Tri-Agency of Canada. VD is the recipient of two Canada Foundation for Innovation grants (37392 and 37858) which supported this work. Computing was performed on Compute Canada infrastructure (RRG2734).

## Acknowledgments

We wish to acknowledge the efforts of Ida Sogaard Larsen who aided in completion of OGTTs and Bruno Marcotte who aided in completion of Bioplex analysis.

## Conflict of interest

The authors declare that the research was conducted in the absence of any commercial or financial relationships that could be construed as a potential conflict of interest.

## Publisher’s note

All claims expressed in this article are solely those of the authors and do not necessarily represent those of their affiliated organizations, or those of the publisher, the editors and the reviewers. Any product that may be evaluated in this article, or claim that may be made by its manufacturer, is not guaranteed or endorsed by the publisher.
