# Supplementary material for: Hemp seed significantly modulates the endocannabinoidome and produces beneficial metabolic effects with improved intestinal barrier function and decreased inflammation in mice under a high-fat, high-sucrose diet as compared with linseed
Source: Front Immunol. 2022 Sep 26;13:882455. doi: 10.3389/fimmu.2022.882455 (PMC9552265; doi:10.3389/fimmu.2022.882455)
Supplement: Supplementary file 1 [file DataSheet_1.docx]

Suppl. Figure 1: Experimental model

Suppl. Figure 2: diet composition.

Suppl. Figure 3: (A) Body composition at week 0; (B) body composition at week 8 corrected by total body weight. ** (p <0.01); *** (p <0.001)

Suppl. Figure 4: Gene expression of *Cidea* and *Pparg1a* in adipose tissue. * (p <0.05) ** (p <0.01); *** (p <0.001)

Suppl. Figure 5: Gene expression of *Acc1*, *Atgl, Hsl* and *Scd1* in adipose tissue. ** (p <0.01); *** (p <0.001)


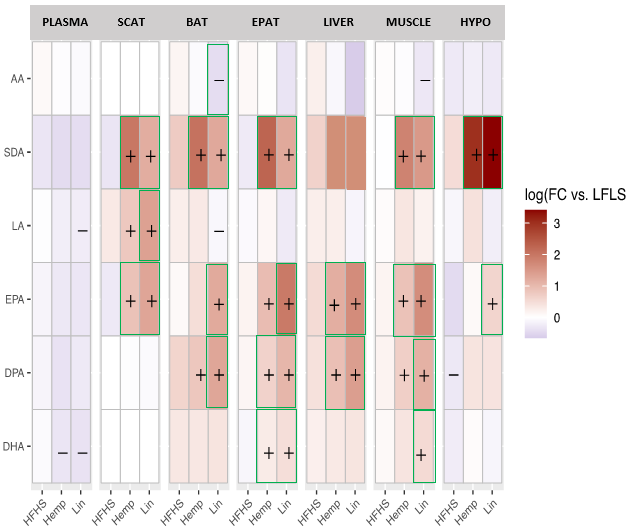


Suppl. Figure 6: Fatty acid levels in different tissues: Brown adipose tissue (BAT), Epididymal adipose tissue (EPAT), Subcutaneous adipose tissue (SCAT), Hypothalamus (HYPO), Liver and Muscle. Statistically significant differences between the high fat high sucrose groups (HFHS, Hemp or Lin) vs the LFLS group are indicated with a ‘’+’’ (increase in expression) or ‘’-‘’ (decrease in expression); p <0.05. Green boxes indicate significant differences between Hemp or Lin and HFHS; p <0.05.


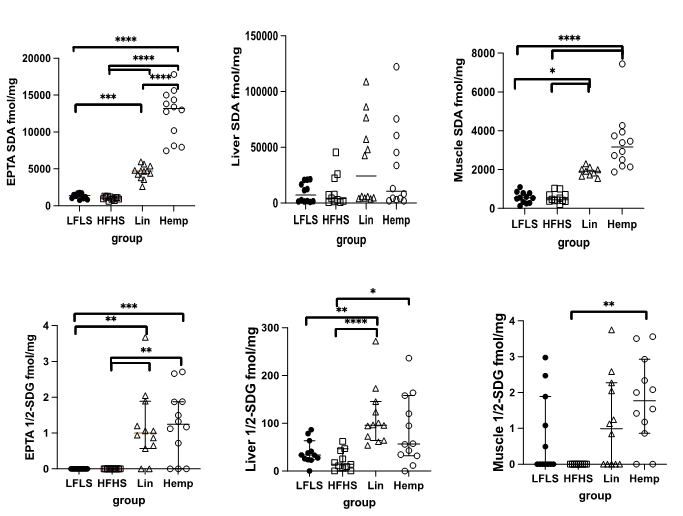


Suppl, Figure 7: SDA and 1/2 SDG profile in selected tissues. * (p <0.05); ** (p <0.01); *** (p <0.001); ****(p <0.00001).


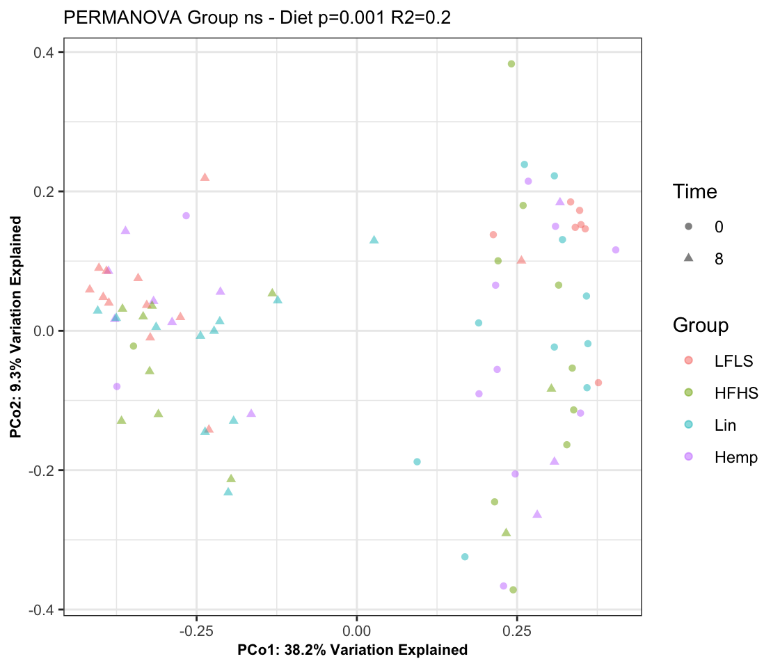

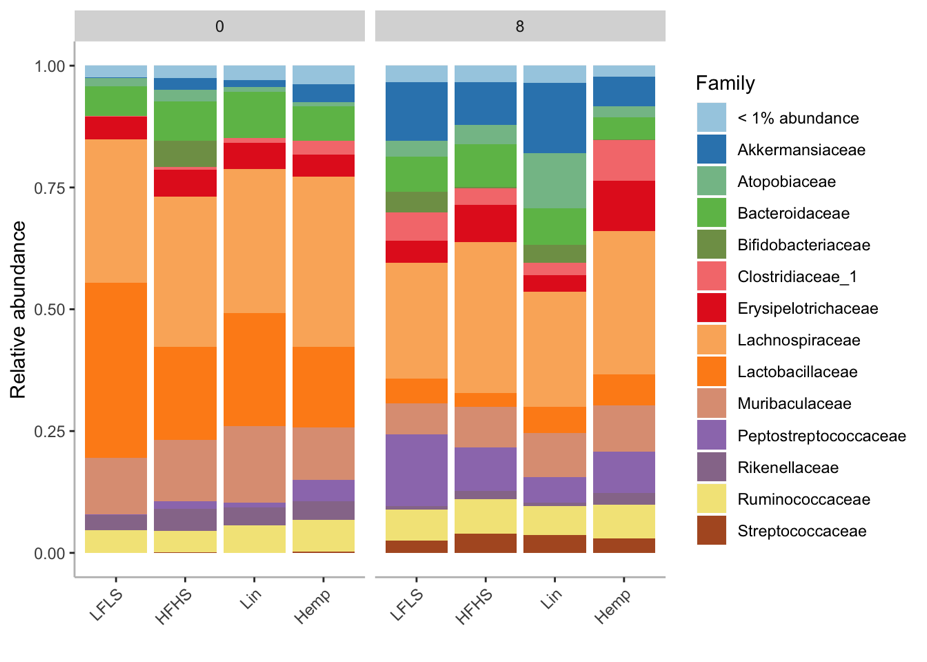


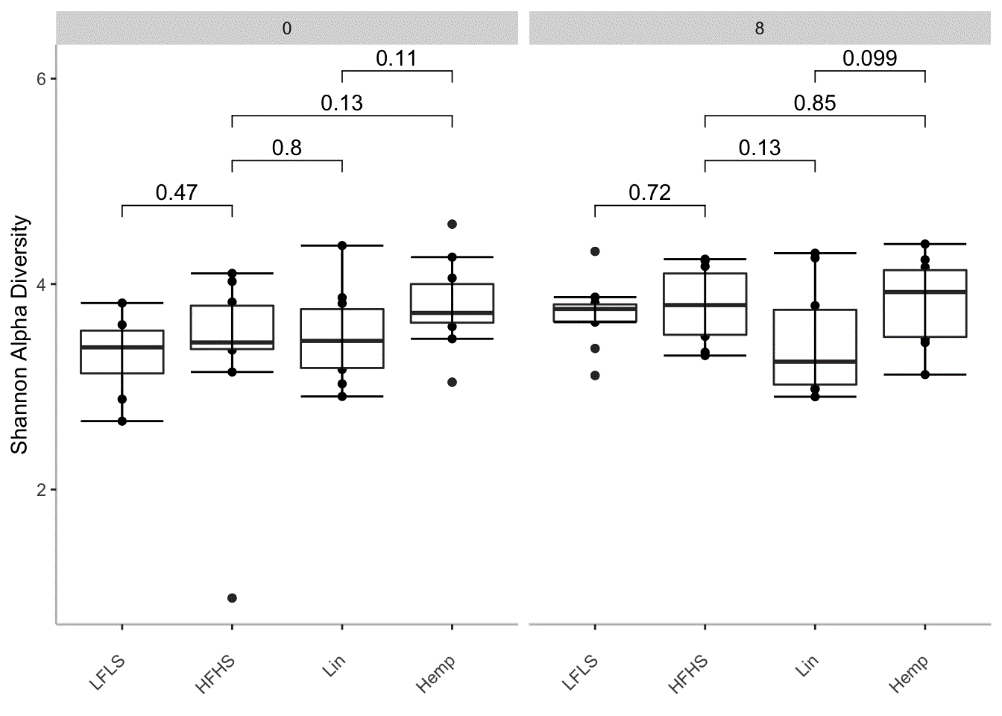

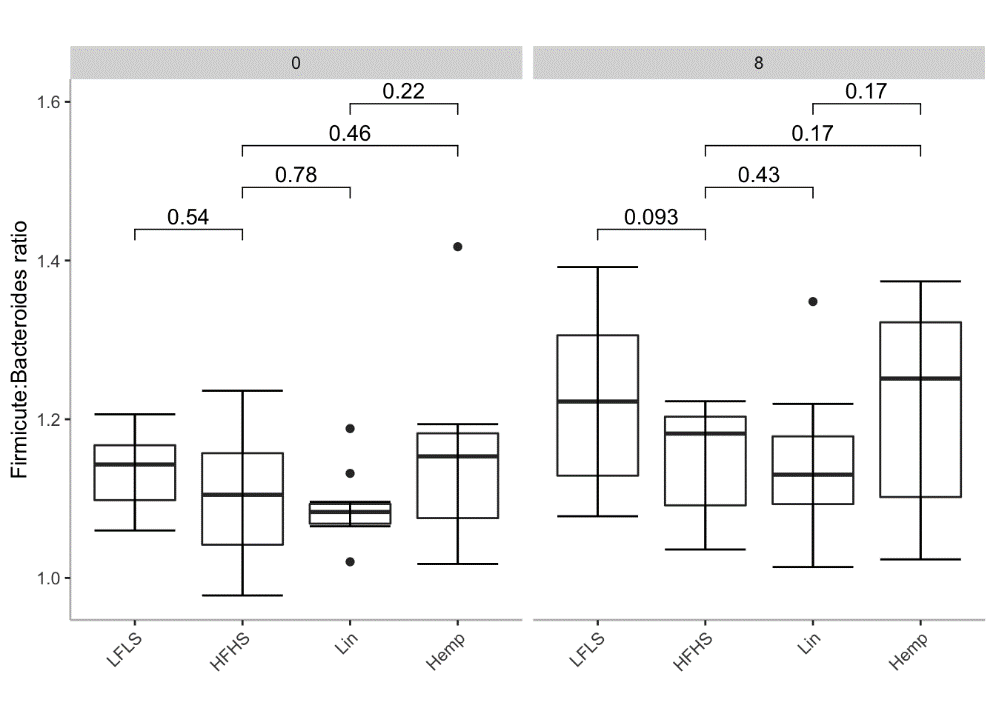


Suppl. Figure 8: Metagenomic analysis: PCoA of all groups showing the effect of switching from chow diet (week 0) to different HFHS diets (week 8) (top left), relative abundance plots of the most prevalent family taxa in each group at (top right), Shannon alpha diversity index to measure bacterial evenness and richness (bottom left) and Firmicutes:Bacteroides ratio (bottom right) at time 0 or after 8 weeks on the HFHS diets.


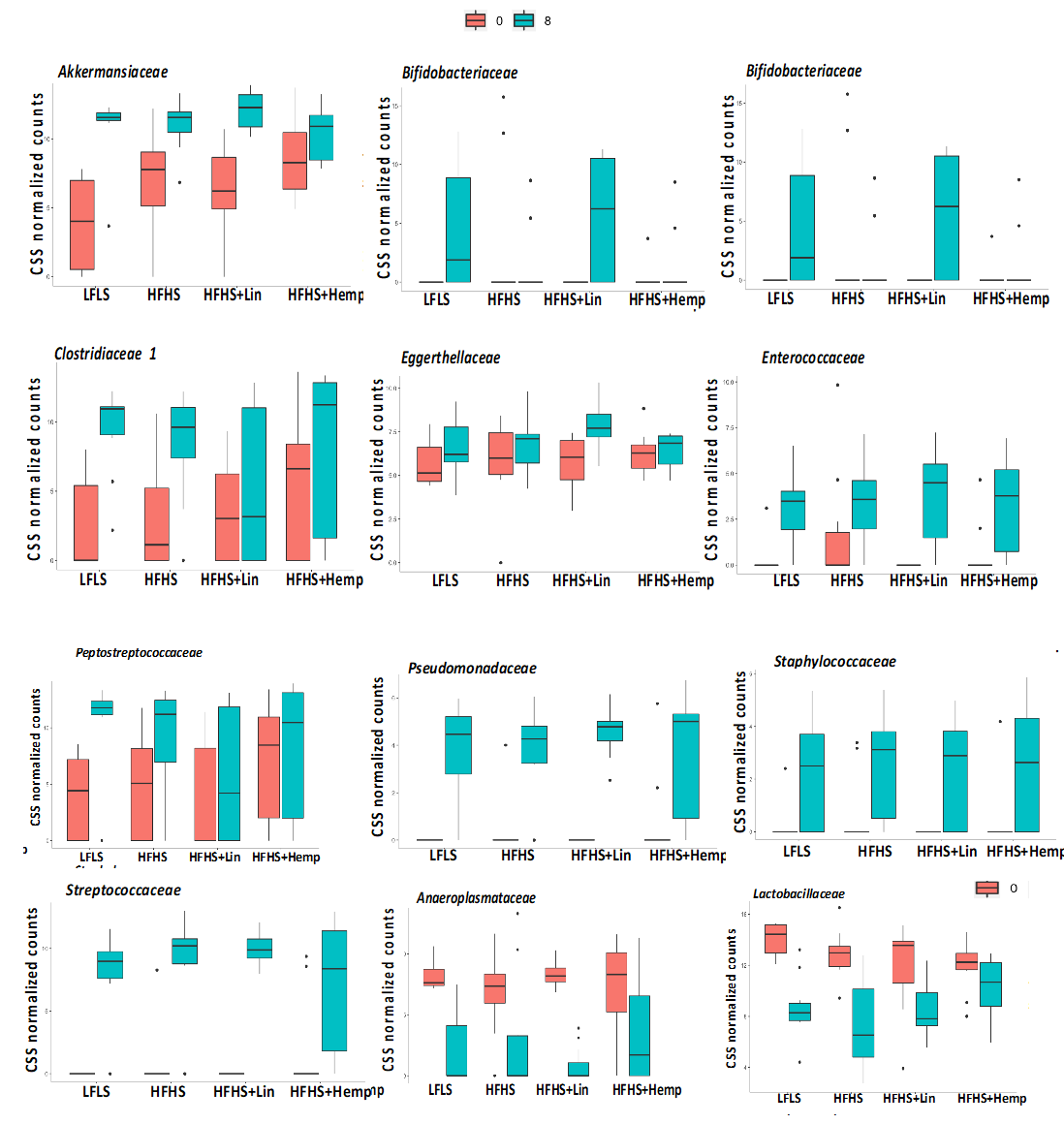

Suppl. Figure 9: Visualization of individual family taxa modified by HFHS diets (top), table detailed results of two-way ANOVA (taxa – Group*Time) followed by Tukey post-hoc analysis showing p-values for intra-diet comparisons between time 0 and week 8 (bottom)

Suppl. Figure 10: Visualization of individual family taxa with different abundances between different diets at week 8 (top), table detailed results of one-way ANOVA followed by Tukey post-hoc analysis showing p-values for inter-diet comparisons at week 8 (bottom)

**Suppl. Table  1:** List of lipid mediators used as internal standards for LC/MS-MS analyses

| **Lipid mediators symbol** | **Lipid mediators name** |
| --- | --- |
| AEA | d4-Anandamide |
| PEA | d4-*N*-palmitoyl-ethanolamine |
| SEA | stearoyl ethanolamide |
| OEA | d4-*N*-oleoyl-ethanolamine |
| LEA | d4-*N*-linoleoyl-ethanolamine |
| EPEA | d4-*N*-eicosapentaenoyl-ethanolamine |
| DPEA.n.3 | docosapentaenoyl ethanolamide |
| DHEA | d4-*N*-docosahexaenoyl-ethanolamine |
| DPEA.n.6 |  |
| 1.2-AG | d5-Mono-arachidonoyl-glycerol 1(3) and 2 isomens |
| 1.2-PG | Mono-palmitoyl-glycerol 1(3) and 2 isomers |
| 1.2-OG | 2-oleoyl-glycerol |
| 1.2-LG | d5-Mono-linoleoyl-glycerol 1(3) and 2 isomers |
| 1.2-SDG | stearidonoyl-glycerol |
| 1.2-EPG | d5-Mono-eicosapentaenoyl-glycerol 1(3) and 2 isomers |
| 1.2-DPG (n-3) | d5-Mono-docosapentaenoyl-glycerol 1(3) and 2 isomers |
| 1.2-DHG | docosahexaenoyl-glycerol |
| AA | d8-Arachidonic acid |
| SDA | stearidonic acid |
| LA | linoleic acid |
| EPA | d5-Eicosapentaenoic acid |
| DPA | d5-Docosapentaenoic acid |
| DHA | d5-Docosahexaenoic acid |
| 13.HODE.G | 13-hydroxyoctadecadienoic acid |
| PGD2 | d4-Prostaglandin D2 |
| PGE2 | d4-Prostaglandin E2 |
| PGF1 | d4-Prostaglandin F1 |
| PGF2 | d4-Prostaglandin F2 |
| Arachidonoyl Serotonin | *N*-Arachidonoyl Serotonin |

**Suppl. Table 2:** List of eCBome genes analyzed by qPCR array

| **Gene symbol** | **Gene name** | **Function** |
| --- | --- | --- |
| *Adgrf1* | adhesion G protein coupled receptor F1 | receptor |
| *Cacna1b* | calcium channel, voltage-dependent, T type, alpha 1B subunit | receptor |
| *Cacna1h* | calcium channel, voltage-dependent, T type, alpha 1H subunit | receptor |
| *Cnr1* | cannabinoid receptor 1 | receptor |
| *Cnr2* | cannabinoid receptor 2 | receptor |
| *Gpr119* | G protein-coupled receptor 119 | receptor |
| *Gpr18* | G protein-coupled receptor 18 | receptor |
| *Gpr55* | G protein-coupled receptor 55 | receptor |
| *Ppara* | peroxisome proliferator activated receptor alpha | receptor |
| *Pparg* | peroxisome proliferator activated receptor gamma | receptor |
| *Ptgfr* | prostaglandin F receptor | receptor |
| *Trpa1* | transient receptor potential cation channel, subfamily A, member 1 | ligand-activated channel |
| *Trpm8* | transient receptor potential cation channel, subfamily M, member 8 | ligand-activated channel |
| *Trpv1* | transient receptor potential cation channel, subfamily V, member 1 | ligand-activated channel |
| *Trpv2* | transient receptor potential cation channel, subfamily V, member 2 | ligand-activated channel |
|  |  |  |
| *Trpv4* | transient receptor potential cation channel, subfamily V, member 4 | ligand-activated channel |
| *Abhd4* | abhydrolase domain containing 4 | anabolic enzyme for NAEs |
| *Akr1b3* | aldo-keto reductase family 1, member B3 (aldose reductase) | anabolic enzyme for prostamides, catabolic enzyme for AEA and 2-AG |
| *Fam213b* | family with sequence similarity 213, member B | anabolic enzyme for prostamides,  catabolic enzyme for AEA and 2-AG |
| *Gde1* | glycerophosphodiester phosphodiesterase 1 | anabolic enzyme for NAEs |
| *Gdpd1* | glycerophosphodiester phosphodiesterase domain containing 1 | anabolic enzyme for NAEs |
| *Glyatl3* | glycine-N-acyltransferase-like 3 | anabolic enzyme for N-acyl-glycines |
| *Hrasls5* | HRAS-like suppressor family, member 5 | anabolic enzyme for NAEs |
| *Inpp5d* | inositol polyphosphate-5-phosphatase D | anabolic enzyme for NAEs |
| *Napepld* | N-acyl phosphatidylethanolamine-specific phospholipase D-like enzyme | anabolic enzyme for NAEs |
| *Pla2g10* | phospholipase A2, group X | anabolic enzyme for NAEs |
| *Pla2g4e* | phospholipase A2, group IVE | calcium-dependent N-acyltransferase that generates NAE biosynthetic precursors |
| *Pla2g5* | phospholipase A2, group V | AA-releasing enzyme possibly involved in phospholipid remodeling and hence biosynthesis of eCB precursors |
| *Ptgs2* | prostaglandin-endoperoxide synthase 2 | anabolic enzyme for prostamides, catabolic enzyme for AEA and 2-AG |
| *Ptpn22* | protein tyrosine phosphatase, non-receptor type 22 (lymphoid) | anabolic enzyme for AEA |
| *Ptges* | prostaglandin E synthase | anabolic enzyme for prostamides, catabolic enzyme for AEA and 2-AG |
| *Comt* | catechol-O-methyltransferase | catabolic enzyme for *N*-acyl-dopamines |
| *Faah* | fatty acid amide hydrolase | catabolic enzyme for NAEs, primary fatty acid amides, *N*-acyl-taurines and *N*-acyl-glycines |
| *Naaa* | N-acylethanolamine acid amidase | catabolic enzyme for saturated NAEs |
| *Pam* | peptidylglycine alpha-amidating monooxygenase | anabolic enzyme for primary fatty acid amides, catabolic enzyme for *N*-acyl-glycines |
| *Dagla* | diacylglycerol lipase, alpha | anabolic enzyme for 2-acylglycerols |
| *Daglb* | diacylglycerol lipase, beta | anabolic enzyme for 2-acylglycerols |
| *Dgke* | diacylglycerol kinase, epsilon | anabolic/catabolic enzyme for 2-acylglycerols |
| *Enpp2* | ectonucleotide pyrophosphatase/phosphodiesterase 2 | autotaxin- a LysoPLD: produces LPA. |
| *Pla1a* | phospholipase A1 member A | anabolic enzyme for 2-acylglycerols |
| *Plcb1* | phospholipase C, beta 1 | anabolic enzyme for 2-acylglycerols |
| *Abhd12* | abhydrolase domain containing 12 | catabolic enzyme for monocylglycerols |
| *Abhd16a* | abhydrolase domain containing 16 | catabolic enzyme for monocylglycerols |
| *Abhd6* | abhydrolase domain containing 6 | catabolic enzyme for monocylglycerols |
| *Agk* | acylglycerol kinase | catabolic enzyme for monocylglycerols |
| *Alox12* | arachidonate 12-lipoxygenase | catabolic enzyme for AEA and 2-AG |
| *Alox15* | arachidonate 15-lipoxygenase | catabolic enzyme for AEA and 2-AG |
| *Ces1d* | carboxylesterase 1D | catabolic enzyme for monoacylglycerols |
| *Ces2h* | carboxylesterase 2H | catabolic enzyme for monoacylglycerols |
| *Mgll (Magl)* | monoglyceride lipase | catabolic enzyme for monoacylglycerols |
| *Mogat1* | monoacylglycerol O-acyltransferase 1 | catabolic enzyme for monoacylglycerols |
| *Ppt1* | palmitoyl-protein thioesterase 1 | catabolic enzyme for 2-AG |
| *Gapdh* | glyceraldehyde-3-phosphate dehydrogenase | reference gene |
| *Hprt* | hypoxanthine guanine phosphoribosyl transferase | reference gene |
| *Rps13* | ribosomal protein S13 | reference gene |
| *Tbp* | TATA box binding protein | reference gene |
